# *Lactobacillus paragasseri* OLL2809 Improves Depression-Like Behavior and Increases Beneficial Gut Microbes in Mice

**DOI:** 10.3389/fnins.2022.918953

**Published:** 2022-06-28

**Authors:** Narumi Hashikawa-Hobara, Ami Otsuka, Chihiro Okujima, Naoya Hashikawa

**Affiliations:** Department of Life Sciences, Okayama University of Science, Okayama, Japan

**Keywords:** depression, OLL2809, gut microbes, *Akkermansia muciniphila*, social defeat stress

## Abstract

*Lactobacillus paragasseri* OLL2809 is a probiotic bacterial strain isolated from healthy human feces. While OLL2809 has been studied for its immunomodulatory activities, its effect on depressive-like behaviors remains unclear. In this study, we used a mouse model of social defeat stress (SDS) to investigate whether oral administration of OLL2809 ameliorates depressive-like behavior. C57BL6 male mice were administered OLL2809 for 2 weeks following a 4-week period of SDS. Although OLL2809 did not affect serum corticosterone levels, it ameliorated depression-like behaviors, and it induced neurite outgrowth in the hippocampal dentate gyrus. The 16S rRNA amplicon sequence analyses revealed that family level gut microbiota composition was affected by stress and OLL2809 administration. Additionally, *Akkermansia muciniphila*, *Bifidobacterium*, and *Lactobacillus* were significantly increased by OLL2809 treatment. LEfSe analysis suggested that the antidepressive effect of OLL2809 may be mediated by increases in other microorganisms, such as Erysipelotrichaceae uncultured. Our findings suggest that *L. paragasseri* OLL2809 may have potential in microbiome therapeutics.

## Introduction

Depression is a highly prevalent mental disorder that severely reduces the quality of life of affected individuals, and severe depressive disorder is associated with high rates of suicide. The prevalence of depressive disorder continues to increase by 18.4% between 2005 and 2015 (World Health Organization [WHO], 2017). Despite the availability of antidepressant treatment for major depression, most patients relapse, with critical impairment, limiting work productivity (Post, 1992; Burcusa and Iacono, 2007). Furthermore, studies suggest that childhood and adolescent antidepressant treatments increase, instead of reducing, suicidal thinking and behavior (Clark et al., 2012). Therefore, the development of new treatments for depression is greatly needed. Notably, it has been reported that gut microbiota is involved in the pathogenesis of depressive disorder, and that probiotics are useful for the prevention of depression (Liu et al., 2020).

It has been speculated that there is a correlation between gastrointestinal (GI) dysfunction and depression symptoms (Mayer et al., 2001; Mussell et al., 2008). Accumulating evidence shows the involvement of the microbiota–gut–brain axis in depressive disorder, which has accordingly become an important research topic (Fung et al., 2017; Liang et al., 2018; Israelyan et al., 2019). In a mouse model of depression, 16s rRNA amplicon sequence analyses and liquid chromatography-mass spectrometry-based metabolomics revealed changes in gut microbiota and fecal metabolic phenotypes that correlate with depression (Kiecolt-Glaser et al., 2015). Furthermore, one study has reported that mice transplanted with the gut microbiome from major depressive disorder patients exhibit depressive-like behavior (Kelly et al., 2016). Another study has reported that large social networks affect the diversity of the gut microbiome composition, which is related to personality traits, in humans (Johnson, 2020).

Two bacterial genera have been the focus of recent studies on depressive disorder: *Bifidobacterium* and *Lactobacillus*. A clinical study has showed that major depressive disorder patients have less *Bifidobacterium* or *Lactobacillus* (Aizawa et al., 2016). In an experimental mouse lipopolysaccharide-induced depressive model, treatment with *Lactobacillus delbrueckii* inhibited depression-like behavior (Qiu et al., 2021). Previous studies revealed that *Lactobacillus paragasseri* OLL2809 (formerly classified as *L. gasseri*) (Tanizawa et al., 2018) can induce an immune reaction (Sashihara et al., 2006), and modulate anxiety and stress in young athletes (Sashihara et al., 2013). Therefore, we hypothesized that OLL2809 may modulate gut microbiota and influence host behaviors and hippocampal neurite outgrowth. To test this, we generated a mouse model of depressive disorder by exposing the animals to a 4-week period of chronic social defeat stress (SDS). We then examined the effects of 2-week oral treatment with OLL2809 on basal serum corticosterone levels and changes in the expression of granule neuron development using doublecortin (DCX) labeling in the hippocampus. We also analyzed alterations in gut microbiota in these mice.

## Materials and Methods

All methods were performed in accordance with the relevant guidelines and regulations.

### Animals

All animal procedures were performed as previously described (Hashikawa-Hobara et al., 2021a). This study was approved by the Animal Care and Use Committee of the Okayama University of Science, and were in accordance with the ARRIVE guidelines. In accordance with these guidelines, efforts were made to minimize the number of animals used and their suffering. All procedures were approved by the institutional ethics committee. Male adult 6-week-old C57BL/6J mice and CD1 (ICR) mice were purchased from Japan SLC Inc. (Shizuoka, Japan). At first, mice were randomly divided into two groups: non-stress control mice and stress mice. Mice were housed in groups of five to six per cage (20 cm × 30 cm × 15 cm, width × length × height), under a 12-h light cycle (lights on at 7:00 AM) at a controlled ambient temperature of 22°C, with 50 ± 10% relative humidity, and with food and water available *ad libitum*. Eight-week-old C57BL/6J mice were used in this experiment. A total of 72 mice were used. Considering the effect of sucrose on the intestinal microorganisms, the experiment was divided into a behavioral test (27 C57BL/6L mice and 18 ICR mice) and fecal microbiota analysis (17 C57BL/6J mice and 10 ICR mice). Because the behavioral test showed variations among mice, the group was set at nine mice. The biochemical parameters were assessed in approximately six animals per group. Histochemical analysis was performed on three animals per group. The fecal assays were done on approximately six animals per group.

### Social Defeat Stress Model

Social defeat stress was performed with some modifications based on our previous research (Hashikawa-Hobara et al., 2021b). Briefly, mice were attacked by ICR mice, which are larger than C57BL/6J. After a 10-min attack, a perforated acrylic plate was placed between the two mice, providing olfactory and visual stimuli for 24 h. This procedure was continued for 4 weeks as exposure to stress. C57BL6J mice were placed in different cages of ICR mice daily to expose them to stress to prevent them from becoming accustomed to each other. If the C57BL6J mice were injured by the ICR mouse attack, they were not given analgesic treatment (to stress them). However, the experiment was discontinued if the attacked mouse lost weight (20% loss in a few days) or was significantly weakened. Two mice dropped out due to injury. After the stress paradigm, mice were divided into the stress sensitivity and resistance groups by social interaction test, which was previously performed in our lab (Hashikawa-Hobara et al., 2021b). Mice were divided into two groups so that the average social index (SI) scores were equal, using their social index with a cutoff value at SI = 1.0, and OLL2809 or vehicle was administered. OLL2809 was mixed with chow and administered for 2 weeks in a group-housed setup (stress: 14 mice, stress + OLL2809: 14 mice). Similarly, the control group was single-housed during stress exposure, and during OLL2809 treatment, control mice were group-housed (control: 16 mice). After bacterial treatment, the forced swim test was performed, followed by the sucrose preference test. Feces were collected on the final day of OLL2809 administration prior to the behavioral test.

### Microorganisms

*Lactobacillus paragasseri* OLL2809, which is reported fatigue-alleviation effect in young athlete (Sashihara et al., 2013), is deposited in the Biological Resource Center, NITE (NBRC; Chiba Japan), with accession number NITE BP-72, was prepared as previously described (Sashihara et al., 2006). Briefly, it was cultured in Lactobacilli Man, Rogosa and Sharpe broth (Fujifilm Wako Pure Chemical Co., Tokyo, Japan) at 37°C for 18 h under anaerobic conditions using Anaeropack (Mitsubishi Gas Chemical, Tokyo, Japan). The dose of *L. paragasseri* OLL2809 was referred as previously reported (Itoh et al., 2011). For treatment, 2 × 10^9^ cfu/day of OLL2809 was administered, after mixing with chow and half the amount of tap water to form dumplings. This dumpling preparation was administered for 19 days from the end of stress exposure to the end of the sucrose preference test. The dumplings were replaced with new ones every 2 days. *Bifidobacterium longum* JCM 1217*^T^* (*B. longum*), *L. paragasseri* JCM 1131*^T^* and *Akkermansia muciniphila* ATCC BAA-835 were purchased from Riken BRC (Wako, Japan). *B. longum* was cultured in Gifu anaerobic medium (Nissui, Tokyo, Japan) as described above. *A. muchiniphila* was cultured anaerobically using a vinyl anaerobic chamber (Coy Laboratory Products; Grass Lake, MI, United States) in Gifu anaerobic medium for 2 days.

### Behavioral Assessments

Open field test was performed as previously described (Hashikawa-Hobara et al., 2021b). Briefly, mice were placed in the center of a square plastic box (30 cm × 40 cm × 20 cm, width × length × height) and allowed to move freely for 3 min. All animal behaviors were monitored and analyzed using Any-Maze behavior tracking software (Muromachi Kikai Co., Ltd., Tokyo, Japan). The open field box was cleaned with 70% ethanol after each test. The test was started from 10:00 AM.

### Forced Swim Test

Forced swim test was performed as previously described (Hashikawa-Hobara et al., 2019). Mice were gently placed in the tank and allowed to swim freely for 6 min. During the test, immobility time was tracked, and the time from the second minute to the end of the test was analyzed using Any-Maze behavior tracking software (Muromachi Kikai Co., Ltd., Tokyo, Japan) The test was carried out from 1:00 PM, after the open field test. The interval between the open field test and the forced swim test was 3 h.

### Sucrose Preference Test

Sucrose preference test was performed as previously described (Hashikawa-Hobara et al., 2021b). Mice were trained for sucrose taste (2% solution) prior to testing. A two-bottle sucrose preference test training was started immediately after the forced swim test. We evaluated mice fluid intake amount between 10:00 AM and 4:00 PM.

### Serum Corticosterone

After behavioral assessment, blood samples were collected from the caudal vein, as previously described (Hashikawa-Hobara et al., 2019). Blood samples were collected from 4:00 PM on Day 48 before brain fixation. Serum corticosterone was measured using an ELISA kit (Assaypro LLC., Saint Charles, MO, United States) in accordance with the manufacturer’s instructions.

### Doublecortin Labeling

After the blood samples has been taken, brains were collected and immunostaining was performed, as previously described (Hashikawa-Hobara et al., 2019). Briefly, the mice were euthanasia by an intraperitoneal administration of sodium pentobarbital (100 mg/kg) and transcardially perfused with saline and 4% paraformaldehyde with 0.35% glutaraldehyde in 0.1 M sodium phosphate buffer. Brains were cut on a cryostat (20 μm), and immunostaining was performed on free-floating sections. Rabbit polyclonal anti-DCX primary antibody (1:1,000; Abcam, Cambridge, United Kingdom) and Alexa Fluor 488 goat anti-rabbit IgG secondary (1:1,000; Life Technologies, Tokyo, Japan) were used. Images were acquired using a fluorescence microscope (FV3000, OLYMPUS, Tokyo, Japan) in the Research Instruments Center of Okayama University of Science. All images of DCX-positive area were measured by Image J software (National Institutes of Health, Bethesda, MD, United States)^1^.

### DNA Extraction From Fecal Samples

Fecal samples were collected from each mouse on day 42. Since sucrose could affect gut bacteria, feces were collected before the sucrose preference test. DNA was extracted as described elsewhere with slight modifications (Costea et al., 2017). Briefly, a total of 20 mg of each sample was bead-homogenized using FastPrep-24 5G (MP Biomedicals, Irvine, CA, United States) with Zirconia beads (EZ-Extract for DNA/RNA, AMR, Tokyo, Japan). Then, DNA was extracted with a Maxwell RSC48 automated nucleic acid extraction apparatus, with a Maxwell RSC PureFood GMO and Authentication kit (Promega, Madison, WI, United States), following the manufacturer’s instructions.

### The 16S rRNA Amplicon Analysis

Bacterial 16S ribosomal RNA (rRNA) gene sequencing was performed according to the MiSeq System Quick Reference Guide^2^. The V3–V4 region of the 16S rRNA gene was amplified by PCR with universal bacterial primer sets (5′-TCGTCGGCAGCGTCAGATGTGTATAAGAGACAGCCTACG GGNGGCWGCAG-3′ and 5′-GTCTCGTGGGCTCGGAGA TGTGTATAAGAGACAGGACTACHVGGGTATCTAATCC-3′) with Illumina adaptor overhang sequences. The amplified libraries were purified using AMPure XP beads (Beckman-Coulter, Indianapolis, IN, United States) as per the manufacturer’s instructions, and eluted with 10 mM Tris–HCl (pH 8.5). The amplicons were sequenced using an Illumina Miseq platform with a Miseq Reagent kit v3 following the manufacturer’s instructions (600 cycles; Illumina, San Diego, CA, United States). Sequence reads were demultiplexed using the Quantitative Insight Into Microbial Ecology (QIIME) 2 version 2019.10^3^ (Bolyen et al., 2019), and errors were corrected with the DADA2 algorithm (Callahan et al., 2016). The taxonomy of the sequence variants was assigned using a feature-classifier plugin that was trained with the taxonomy information in majority_taxonomy_all_levels.txt of 99% clustering in SILVA version 132^4^ (Quast et al., 2013). We rarefied the data to 25,000 sequences per sample to conduct several analyses.

### Quantitative Analysis by Real-Time PCR

A 4-μL aliquot of DNA solution was mixed with 10 μL of PowerUP SYBER Green Master Mix (Applied Biosystems, Waltham, MA, United States), 0.18 μL each of forward and reverse primer solutions (100 pmol/μL) and 5.64 μL MilliQ water to a total volume of 20 μL. Real-time PCR was performed using QuantStudio 3 Real-time PCR system (Applied Biosystems). The PCR reaction consisted of one cycle of 50°C for 2 min and 95°C for 2 min, followed by 40 cycles of 95°C for 15 s and 60°C for 1 min. The primer sets are shown in Supplementary Table 1. The standard curves were prepared using the following bacteria: *B. longum* JCM 1217*^T^*, genus *Lactobacillus*, *L. gasseri* JCM 1131*^T^*, and *A. muciniphila* ATCC BAA-835. A melting curve analysis was carried out to ensure the specificity of the amplification products.

### Statistical Analysis

All data are expressed as the mean ± S.E.M. Because the investigators only handled the numbering of the mice, they were blinded to treatment during data analysis. The data were analyzed using the Tukey–Kramer test or Dunn’s test. Correlation analysis between immobility time in the forced swim test and gut microbial abundances was performed with Spearman correlation test. The statistical analyses were performed using GraphPad Prism 9 software (GraphPad Software Inc., San Diego, CA, United States). Beta diversity of the gut microbiota was analyzed using the Jaccard method to identify compositional differences (Lozupone et al., 2011). PERMANOVA was used to test the significance among groups. Significant differences in microbial taxa abundance between two groups was analyzed using LEfSe^5^ (Segata et al., 2011). A *p*-value < 0.05 was considered statistically significant.

## Results

### Effects of 2-Week Treatment With OLL2809 on Depressive Behavior After Chronic Stress Exposure

**FIGURE 1:**
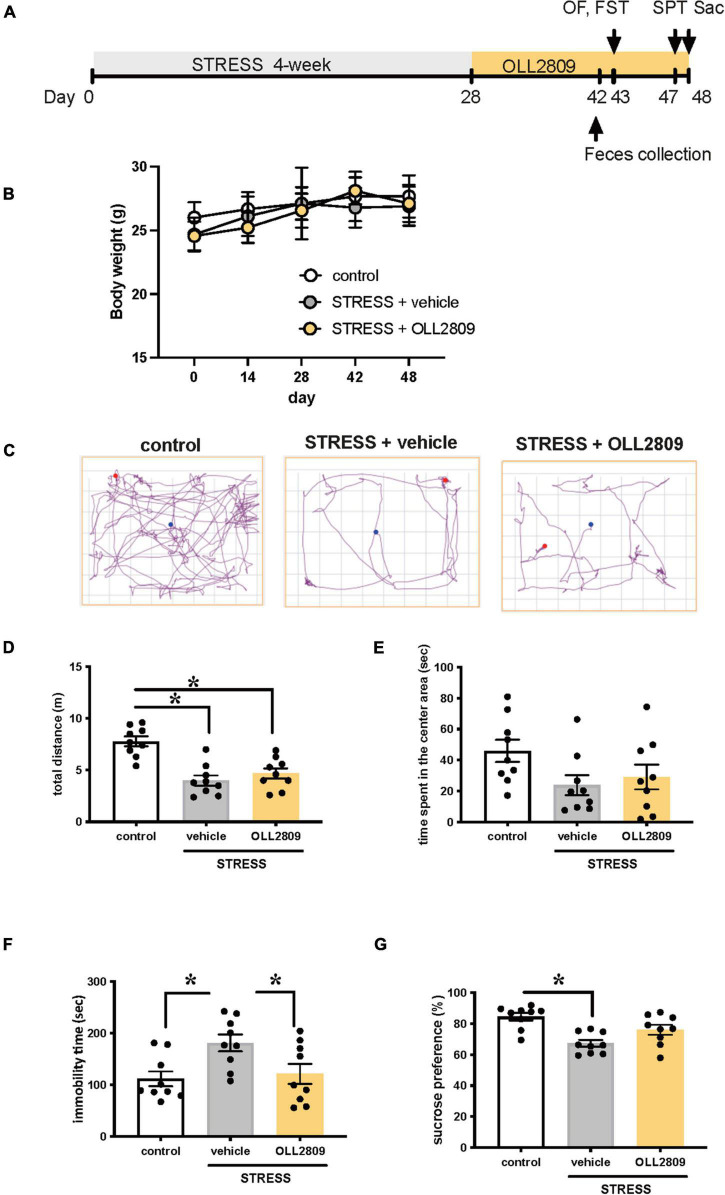
*Lactobacillus paragasseri* OLL2809 rescues depression-like behavior. **(A)** Outline of the experimental protocol. **(B)** Body weight. **(C)** Representative motion tracks for control, stress + vehicle and stress + OLL2809 groups in the open field test. **(D)** Locomotion: total distance travel in the open field test. **(E)** Time spent in the center area in the open field test. **(F)** Immobility times in the forced swim test. **(G)** The percentage of sucrose preference in the sucrose preference test. Each bar indicates the mean ± S.E.M. *p < 0.05, Tukey–Kramer test. Each plot represents the number of individual mice. Control, *n* = 9; stress + vehicle, *n* = 9; stress + OLL2809, *n* = 9; OF, open field test; FST, forced swim test; SPT, sucrose preference test; Sac, sacrificed.

To investigate whether OLL2809 alleviates depression-like behavior, mice were given OLL2809 mixed with chow for 20 days after 4 weeks of stress exposure (Figure 1A). We first examined whether body weight was affected by stress or OLL2809 treatment. There were no changes in body weight among the groups throughout the experimental period (Figure 1B). After 14 days of oral OLL2809 treatment, on day 43, we performed the open field test. We recorded 3 min of general behavior, and analyzed locomotor activity and time spent in the center area (Figures 1C–E). Stressed mice showed significantly reduced total distance (locomotor activity), compared with control animals (control vs. vehicle, *p* < 0.0001; control vs. OLL2809, *p* = 0.0004; Figure 1D). OLL2809 did not improve stress-mediated decreases in locomotor activity (Figure 1D). Time spent in the center area, which is considered an index of anxiety, did not significantly differ among the three groups (Figure 1E). Next, to examine whether OLL2809 affects depression-like behavior, we performed the forced swim test, which is commonly used to analyze depression-like behavior. Even 2 weeks after the 4-week period of stress exposure, an increase in immobility time was still observed, compared with control (control vs. vehicle, *p* = 0.0194; Figure 1F). This prolongation of immobility time was significantly suppressed by OLL2809 administration (vehicle vs. OLL2809, *p* = 0.0471; Figure 1F). Next, we performed the sucrose preference test. Sucrose preference was significantly reduced in stressed mice, but not in stressed mice given OLL2809 treatment (control vs. vehicle, *p* = 0.0004; Figure 1G). These results indicate that OLL2809 exerts antidepressant effects.

### Effects of 2-Week Treatment With OLL2809 on Corticosterone Levels and Cell Proliferation in the Hippocampal Dentate Gyrus After Chronic Stress Exposure

**FIGURE 2:**
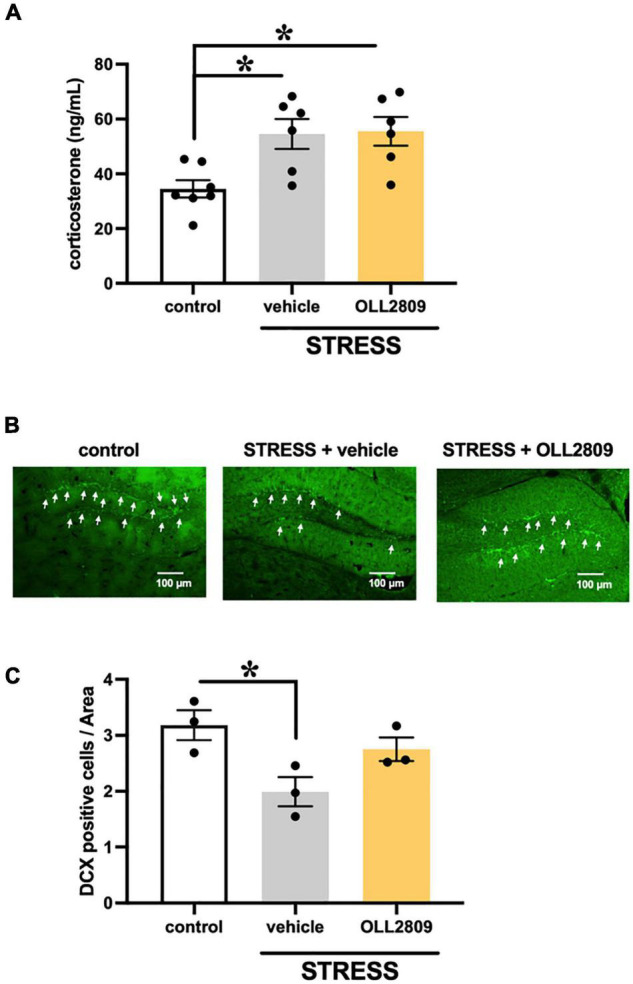
Effects of *Lactobacillus paragasseri* OLL2809 on serum corticosterone levels and cell proliferation in the hippocampal dentate gyrus. **(A)** Serum corticosterone levels. Control, *n* = 7; stress + vehicle, *n* = 6; stress + OLL2809, *n* = 6. **(B)** Photomicrographs of the dentate gyrus showing representative doublecortin (DCX) immunostaining. The scale bar indicates 100 μm. **(C)** The density of the DCX-positive area. Control, *n* = 3; stress + vehicle, *n* = 3; stress + OLL2809, *n* = 3. Each bar indicates the mean ± S.E.M. *p < 0.05, Tukey–Kramer test. Each plot represents the number of mice.

To investigate whether OLL2809 affects serum corticosterone, we measured its levels by ELISA. The corticosterone levels in the stress group were significantly increased compared with the control. Mice given OLL2809 treatment showed no difference compared with the vehicle group (control vs. vehicle, *p* = 0.0172; control vs. OLL2809, *p* = 0.0127; Figure 2A). Next, to determine whether hippocampal neurogenesis is responsible for the OLL2809-mediated antidepressive effect, immunohistochemical analysis was used to evaluate granule neuron development using DCX, a marker of neural progenitors. We observed fewer DCX-positive cells (39.5%) in the hippocampal subgranular zone of the dentate gyrus in the vehicle group compared with control mice (Figure 2B). DCX-positive cells were significantly reduced in stressed mice, but not in mice given 2 weeks of OLL2809 administration (control vs. vehicle, *p* = 0.0341; Figure 2C).

### Alterations in the Gut Microbiota Induced by Chronic Stress and *Lactobacillus paragasseri* OLL2809 Treatment

**FIGURE 3:**
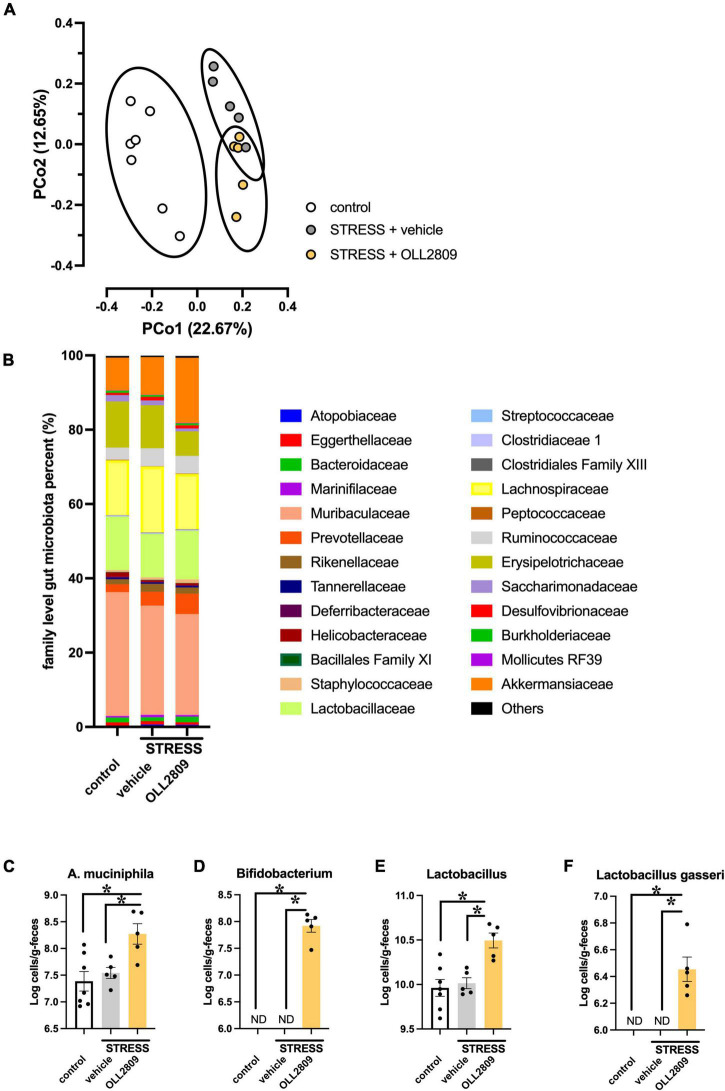
The 16S rRNA amplicon sequence analysis reveals changes in mouse gut microbial diversity and the relative abundance of microorganisms. **(A)** The PCoA analysis plots of Jaccard-based diversity. Each circle represents 95% confidence interval of the group. **(B)** Relative abundance of gut microorganisms at the family level. **(C)** Quantitative real-time PCR analysis of *Akkermansia muciniphila*, **(D)** *Bifidobacterium*, **(E)** *Lactobacillus*, and **(F)** *Lactobacillus gasseri*. Each bar indicates the mean ± S.E.M. *p < 0.05, PERMANOVA, Tukey–Kramer test or Dunn’s test. Each plot represents the number of mice. ND: not detected (detection limit, 6.0 log cells/g feces). Control, *n* = 7; stress + vehicle, *n* = 5; stress + OLL2809, *n* = 5.

OLL2809 treatment had antidepressant-like effects, and therefore we hypothesized that it may alter gut bacterial composition. We thus analyzed the composition of the gut microbiota by 16S rRNA amplicon sequence analyses. The alpha diversity (faith_pd index) showed no significant difference between groups (Supplementary Material). The principal coordinates analysis (PCoA) plots of Jaccard-based diversity among the three groups revealed compositional differences. Significant differences in the composition among all three groups were detected by permutational multivariate analysis of variance (PERMANOVA) (control vs. vehicle, *p* = 0.002; vehicle vs. OLL2809, *p* = 0.004; control vs. OLL2809, *p* = 0.003; Figure 3A). The composition of the gut microbiota, at the family level, in the three groups is shown in Figure 3B. We found that the relative abundances of Akkermansiaceae were increased by OLL2809 treatment after stress exposure, although stress exposure alone did not affect the levels (Figure 3B). The relative abundances in the control, vehicle and OLL2809 groups were 8.8, 10.2, and 17.6%, respectively. We focused on *Lactobacillus*, which is generally reduced by stress exposure (Aizawa et al., 2016). We found that stress exposure decreased *Lactobacillus* percentage in the gut microbiota. The percentages in the control, vehicle and OLL2809 groups were 14.4, 11.7, and 13.1%, respectively. We performed quantitative real-time PCR to quantify these microbes. *Akkermansia muciniphila* was significantly increased in the OLL2809 group compared with the control group, while there was no difference between the control and vehicle groups. The mean numbers in the control, vehicle and OLL2809 groups were 7.38, 7.54, and 8.27 log cells/g, respectively (control vs. OLL2809, *p* = 0.0063; vehicle vs. OLL2809, *p* = 0.0341; Figure 3C). We also examined *Bifidobacterium* because of its reported involvement in depressive-like behavior (Arseneault-Bréard et al., 2012) (included in Others in Figure 3B because of its quite low relative abundances). While it was not detected in any mouse in the control and stress groups, OLL2809 administration significantly increased *Bifidobacterium* (Figure 3D). *Bifidobacterium* was under the detection limit (6.0 log cells/g feces) in all mice in both the control and vehicle groups, but was detected in all mice in the OLL2809 group, as 7.92 log cells/g, which was at least 84-fold higher than in the other groups. Because of this limitation, the value of each mouse in the control and vehicle groups was set to 6.0, and statistical testing was performed non-parametrically, using Dunn’s test. The number was remarkably increased in the OLL2809 group (control vs. OLL2809, *p* = 0.0008; vehicle vs. OLL2809, *p* = 0.0013; Figure 3D). We also examined whether OLL2809 affected the genus *Lactobacillus* using quantitative real-time PCR. Unlike the result by 16S rRNA amplicon sequence analyses (Figure 3B), stress exposure did not change the relative amount of *Lactobacillus*, but OLL2809 significantly increased it (control vs. OLL2809, *p* = 0.0016; vehicle vs. OLL2809, *p* = 0.0064; Figure 3E). Next, to clarify whether the increase in *Lactobacillus* in the OLL2809 group was caused by orally administered *L. paragasseri* OLL2809, we analyzed the number of *L. gasseri* (including *L. paragasseri*) using species-specific primers. *L. gasseri* was not detected in any mouse in either the control or vehicle group (detection limit was 6.0 log cells/g feces) (control vs. OLL2809, *p* = 0.0008; vehicle vs. OLL2809, *p* = 0.0013; Figure 3F). Although the number of *L. gasseri* was significantly increased, 6.45 log cells/g, in the OLL2809 group, it was less than 0.01% of the genus *Lactobacillus* detected in the OLL2809 group, indicating that the increase in *Lactobacillus* in the OLL2809 group was not caused by *L. paragasseri* OLL2809.

### Bacterial Taxa Differences Among the Groups Assessed by Linear Discriminant Analysis Effect Size Tables

**FIGURE 4:**
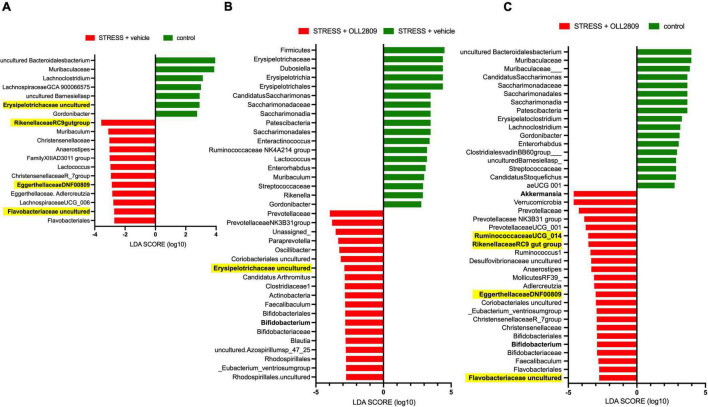
Taxonomic differences in gut microbiota among control, stress + vehicle and stress + OLL2809 groups using linear discriminant analysis (LDA) effect size (LEfSe) analysis (LDA score > 2). **(A)** Control-enriched taxa are indicated with a positive LDA score (green), and taxa enriched by stress have a negative score (red). **(B)** Stress-enriched taxa are indicated with a positive LDA score (green), and taxa enriched in stress + OLL2809 mice have a negative score (red). **(C)** Control-enriched taxa are indicated with a positive LDA score (green), and taxa enriched in stress + OLL2809 mice have a negative score (red). Bacteria highlighted in yellow are correlated significantly with immobility time in the forced swim test. Control, *n* = 7; stress + vehicle, *n* = 5; stress + OLL2809, *n* = 5.

Next, we performed LEfSe analysis to detect gut microorganisms significantly affected by stress and OLL2809 treatment. The relative abundances of the 19 operational taxonomic units (OTUs), including Erysipelotrichaceae uncultured (LDA = 2.91, *p* = 0.0043), Rikenellaceae RC9 gut group (LDA = −3.57, *p* = 0.0045), Eggerthellaceae DNF0089 (LDA = −2.88, *p* = 0.0159) and Flavobacteriaceae uncultured (LDA = −2.72, *p* = 0.0042), were different in the vehicle group compared with the control group (Figure 4A). Furthermore, 37 OTUs, including *Bifidobacterium* (LDA = −2.84, *p* = 0.0053) and Erysipelotrichaceae uncultured (LDA = −2.89, *p* = 0.0086), were different in the OLL2809 group compared with the vehicle group (Figure 4B). When comparing the OLL2809 group with the control group, 40 OTUs, including *Akkermansia* (LDA = −4.61, *p* = 0.0424), *Bifidobacterium* (LDA = −2.91, *p* = 0.0015), Ruminococcaceae UCG_014 (LDA = −3.55, *p* = 0.0424), Rikenellaceae RC9 gut group (LDA = −3.53, *p* = 0.0185), Eggerthellaceae DNF0089 (LDA = −3.00, *p* = 0.0035) and Flavobacteriaceae uncultured (LDA = −2.75, *p* = 0.0071), were detected as characteristic microorganisms (Figure 4C).

### Correlation Analysis Between Immobility Time in the Forced Swim Test and Gut Microbiota Composition

**FIGURE 5:**
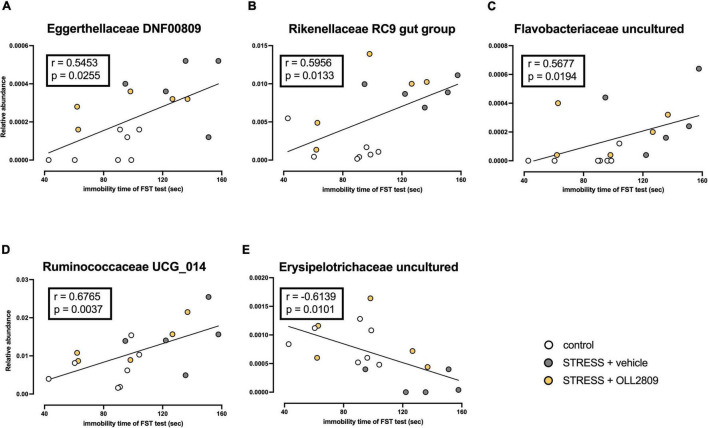
Correlation between immobility time in the forced swim test and the gut microbial abundances for which significant changes were detected by LEfSe analysis. **(A)** Eggerthellaceae DNF00809, **(B)** Rikenellaceae RC9 gut group, **(C)** Flavobacteriaceae uncultured, **(D)** Ruminococcaceae UCG_014 and **(E)** Erysipelotrichaceae uncultured. The Spearman correlation analyses of *R*-values and *p*-values are shown in each box. Control, *n* = 7; stress + vehicle, *n* = 5; stress + OLL2809, *n* = 5.

**FIGURE 6:**
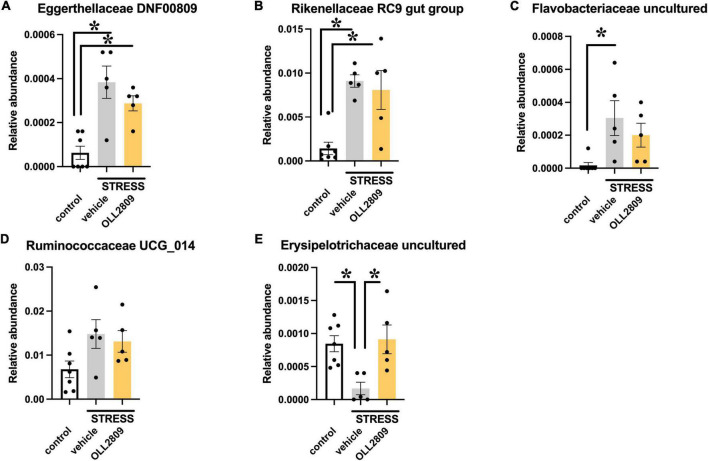
Effects of *Lactobacillus paragasseri* OLL2809 administration on relative abundance of gut microorganisms that showed significant correlation with immobility time in the forced swim test. **(A)** Eggerthellaceae DNF00809, **(B)** Rikenellaceae RC9 gut group, **(C)** Flavobacteriaceae uncultured, **(D)** Ruminococcaceae UCG_014 and **(E)** Erysipelotrichaceae uncultured. Each bar indicates the mean ± S.E.M. *p < 0.05, Tukey–Kramer test. Each plot represents the number of mice. Control, *n* = 7; stress + vehicle, *n* = 5; stress + OLL2809, *n* = 5.

To analyze whether specific microorganisms affect immobility time in the forced swim test, we performed correlation analysis between the relative abundances of the gut microbiota for which significant changes were detected by LEfSe analysis and immobility time in the forced swim test. The analysis revealed a significant positive correlation between immobility time and Eggerthellaceae DNF0089 (*r* = 0.5453, *p* = 0.0255), Rikenellaceae RC9 gut group (*r* = 0.5956, *p* = 0.0133), Flavobacteriaceae uncultured (*r* = 0.5677, *p* = 0.0194) and Ruminococcaceae UCG_014 (*r* = 0.6765, *p* = 0.0037) (Figures 5A–D). In contrast, a significant negative relationship was detected for Erysipelotrichaceae uncultured (*r* = −0.6139, *p* = 0.0101) (Figure 5E). Next, we compared the relative abundances of these microorganisms. We found that both Eggerthellaceae DNF00809 and Rikenellaceae RC9 gut group were significantly increased by stress exposure, regardless of OLL2809 treatment (control vs. vehicle, *p* = 0.0005; control vs. OLL2809, *p* = 0.0093; Figure 6A; control vs. vehicle, *p* = 0.0019; control vs. OLL2809, *p* = 0.0057; Figure 6B). Flavobacteriaceae uncultured was significantly increased by stress exposure, but not by OLL2809 administration (control vs. vehicle, *p* = 0.0191; Figure 6C). Among these, only Ruminococcaceae UCG_014 did not show significant changes (Figure 6D). Erysipelotrichaceae uncultured showed significantly reduced composition in the stress group but was restored to control levels by administration of OLL2809 (control vs. vehicle, *p* = 0.0143; vehicle vs. OLL2809, *p* = 0.0129; Figure 6E). These results suggest that OLL2809 administration may ameliorate depressive behavior in this mouse model of chronic stress-induced depression by increasing the number of Erysipelotrichaceae uncultured.

## Discussion

In the present study, we found that *L. paragasseri* OLL2809 administration exerted antidepressive effects in mice exposed to SDS. OLL2809 elevated the number of *Akkermansia muciniphila*, *Bifidobacterium*, and *Lactobacillus* in feces. Furthermore, by correlating the microorganisms detected by LEfSe analysis with immobility time, we found that Erysipelotrichaceae uncultured may be a new candidate bacterium involved in the antidepressant-like effect of OLL2809.

Corticosterone is one of the key factors involved in depressive behavior. Generally, stress exposure elevates glucocorticoid release from the adrenal cortex and increases arterial tension, with associated elevated blood pressure and blood glucose levels. Chronic stress maintains high levels of glucocorticoids and induces depressive symptoms. Previous studies show that long-term administration of corticosterone produces depression, with disruption of the hypothalamic–pituitary–adrenal (HPA) axis in rodents (Johnson et al., 2006; Hill et al., 2015; Ding et al., 2018). Thus, high corticosterone levels can influence behavioral responses. A recent study showed that antibiotic treatment significantly decreases serum corticosterone in the mouse (Zhang et al., 2020), indicating that there is bidirectional communication between the HPA axis and the gut microbiota. In the present study, serum corticosterone was significantly increased by stress, but was unaffected by OLL2809. In contrast, prior studies showed that *Lactobacillus reuteri* NK33 and *helveticus* NS8 significantly suppress corticosterone levels in mice subjected to immobilization or chronic restraint stress (Liang et al., 2015; Jang et al., 2019). We speculate that the discrepancy from our findings is that the time from stress exposure to blood sampling was as short as 2 weeks. Conceivably, OLL2809 did not affect corticosterone levels in this stress model. However, OLL2809 increased cell proliferation in the hippocampal dentate gyrus, indicating that it may exert antidepressive effects through a mechanism that does not involve corticosterone. Further studies should be conducted to clarify the underlying mechanisms.

Accumulating evidence suggests that alterations in the gut microbiota composition are associated with the pathogenesis of depression (Desbonnet et al., 2010; Friswell et al., 2010; Aizawa et al., 2016; Zheng et al., 2020). In the present study, stress exposure did not change the number of *Lactobacillus*, *Bifidobacterium*, or *A. muciniphila*. Consistent with our results, Yang et al. (2017) reported that *Bifidobacterium* did not affect mice exposed to SDS compared with non-stressed mice. While we did not examine why stress exposure did not change the composition of major bacteria, *Lactobacillus*, *Bifidobacterium*, and *A. muciniphila*, the collection of feces 14 days after stress exposure may be a contributing factor. Notably, OLL2809 treatment significantly increased these microorganisms, suggesting that OLL2809 may activate gut microbiota, accelerate granule neuron development in the hippocampus, and ameliorate depression-like behavior. Although the mechanisms underlying the antidepressive action of OLL2809 remain unclear, an increase in probiotics could have beneficial effects.

We found here that administration of *L. paragasseri* OLL2809 increased the abundances of beneficial microorganisms such as *Akkermansia* and *Bifidobacterium*. Only two species have been reported in the genus *Akkermansia*: *A. muchiniphila* and *A. glycaniphila* (Post, 1992; Belzer and de Vos, 2012; Zhang et al., 2019). *A. glycaniphila* was recently isolated from python feces and has the ability to degrade mucin (Ouwerkerk et al., 2016). *A. muchiniphila* was recently isolated from healthy human fecal samples and has been identified as a mucin-degrading bacteria (Derrien et al., 2004). *A. muchiniphila* plays an important role in the development of some diseases, including type 2 diabetes, atopy, and obesity (Drell et al., 2015; Dao et al., 2016; Chelakkot et al., 2018; Zhang et al., 2019). Notably, *A. muchiniphila* has been reported to be reduced in children with autism (Wang et al., 2011), which is commonly associated with gastrointestinal dysfunctions (Campion et al., 2018) and is closely associated with depression (Magnuson and Constantino, 2011). Although we observed that stress exposure did not induce a decrease in *A. muchiniphila*, treatment with OLL2809 significantly increased his bacterium. The increase in *A. muchiniphila* is possibly a result of the OLL2809-mediated antidepressive effect. Furthermore, we also observed increased levels of *Bifidobacterium* and *Lactobacillus* after OLL2809 treatment. Oral intake of *Bifidobacterium*, a genus of gram-positive anaerobic bacteria, was recently reported to improve depressive behavior in SDS-exposed mice (Yang et al., 2017) and in the maternal separation model (Desbonnet et al., 2010). *Bifidobacterium* reduces intestinal endotoxin levels and improves mucosal barrier function (Griffiths et al., 2004; Pinzone et al., 2012). Additionally, *Lactobacillus* is the major genus used for probiotics, and is reduced in patients with major depressive disorder (Aizawa et al., 2016). The combination of *Lactobacillus* and *Bifidobacterium* has an antidepressive effect when administered to rodents (Arseneault-Bréard et al., 2012). A more recent report suggested that *Lactobacillus* suppresses inflammation, with decreases in toll-like receptor, NOD-like receptor and interleukin-1β, and exerts an antidepressant-like effect (Qiu et al., 2021). Cytokines changes are well known to be implicated in the pathology of depression (Himmerich et al., 2019). *Lactobacillus paragasseri* OLL2809 has been reported that ameliorating action of reduction in natural killer cells and decreasing in the serum reactive oxygen metabolites in young athlete (Sashihara et al., 2013). In the present study, we did not evaluate the effects of OLL2809 on cytokines such as interferon-gamma. Further consideration is needed. We found marked increases in the levels of *Lactobacillus* and *Bifidobacterium* in mice given OLL2809 treatment after chronic stress. These results suggest that the administration of this strain increases the number of other beneficial bacteria, which may underlie its antidepressive effect.

From LEfSe analysis, 19 microbial species were changed in abundance by stress exposure and 37 were changed by administration of OLL2809. Furthermore, the relative abundances of five bacteria were significantly correlated with immobility time in the forced swim test. Among these, only Erysipelotrichaceae uncultured showed a significant difference. Thus, Erysipelotrichaceae uncultured possibly impacts depression-like behavior. Erysipelotrichaceae, which belong to the Firmicutes phylum, have been linked to elevated tumor necrosis factor (TNF)-α levels in patients with chronic HIV infection (Dinh et al., 2015). Lower levels of Erysipelotrichaceae were observed in fecal samples of patients with major depressive disorder (MDD) (Jiang et al., 2015). Conversely, another study reported that Erysipelotrichaceae were higher in MDD patients than in healthy controls (Zheng et al., 2016). The discrepancies may result from differences in the recruitment of participants and the procedure for sample collection. Our results showed that Erysipelotrichaceae and *Dubosiella*, a genus of Erysipelotrichaceae, were different in the stress group compared with the OLL2809 group (Figure 4B). Further investigation is needed to clarify whether the increase in Erysipelotrichaceae protects against depression. Because Erysipelotrichaceae uncultured is difficult to culture, no reports have shown that it is associated with depression. Therefore, it remains unclear whether Erysipelotrichaceae uncultured directly ameliorates depressive behavior. This is a major limitation of this study and further research is required. Nonetheless, our current findings suggest that Erysipelotrichaceae uncultured may be a novel candidate biomarker of depression, and that OLL2809 may exert its beneficial effect by upregulating this bacterial family.

In conclusion, our present findings suggest that probiotics containing *Lactobacillus paragasseri* OLL2809 may increase other beneficial microorganisms, such as *Bifidobacterium*, *Lactobacillus*, and *Akkermansia*, have health promoting effects, and may prevent depression-like behavior in mice. Moreover, Erysipelotrichaceae uncultured was correlated with immobility time in the forced swim test, and OLL2809 increased the levels of this family. Our current findings should further our understanding of the beneficial effects of *L. paragasseri* OLL2809 as a probiotic for microbiome therapeutics.

## Data Availability Statement

The datasets presented in this study can be found in online repositories. The names of the repository/repositories and accession number(s) can be found below: https://ddbj.nig.ac.jp/resource/bioproject/PRJDB13039.

## Ethics Statement

This study involving animals was reviewed and approved by The Animal Care and Use Committee of the Okayama University of Science.

## Author Contributions

NH and NH-H designed the experiments and wrote the manuscript. CO, AO, and NH conducted the experiments. NH-H conducted the statistical analyses and prepared the figures. All authors reviewed and approved the manuscript.

## Conflict of Interest

The authors declare that the research was conducted in the absence of any commercial or financial relationships that could be construed as a potential conflict of interest.

## Publisher’s Note

All claims expressed in this article are solely those of the authors and do not necessarily represent those of their affiliated organizations, or those of the publisher, the editors and the reviewers. Any product that may be evaluated in this article, or claim that may be made by its manufacturer, is not guaranteed or endorsed by the publisher.

## Footnotes

1
http://imagej.nih.gov/ij/

2
https://support.illumina.com/

3
https://qiime2.org

4
https://www.arb-silva.de/download/archive/

5
http://huttenhower.sph.harvard.edu/galaxy/
