# Supplementary figures and images for: Lactobacillus paragasseri OLL2809 Improves Depression-Like Behavior and Increases Beneficial Gut Microbes in Mice
Source: Front Neurosci. 2022 Jun 28;16:918953. doi: 10.3389/fnins.2022.918953 (PMC9274989; doi:10.3389/fnins.2022.918953)

# alpha diversity

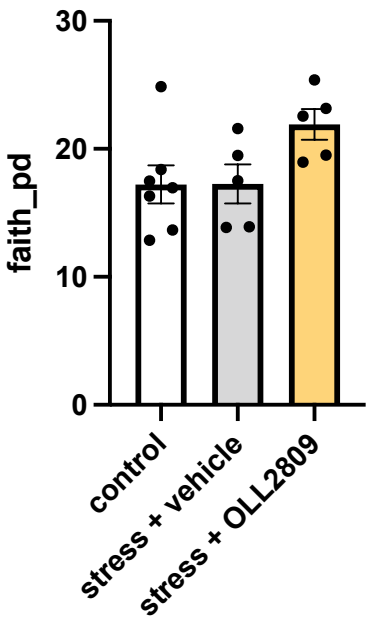

Supplement: Supplementary file 1 [file Data_Sheet_1.PDF]
