# Supplementary material for: Lactobacillus paragasseri OLL2809 Improves Depression-Like Behavior and Increases Beneficial Gut Microbes in Mice
Source: Front Neurosci. 2022 Jun 28;16:918953. doi: 10.3389/fnins.2022.918953 (PMC9274989; doi:10.3389/fnins.2022.918953)
Supplement: Supplementary file 2 [file Table_1.docx]

**Supplementary 1.** Altered gut microbiota in mice. The alpha diversity (fait_pd index) in three groups.

| **Target bacteria** | **primer** | **Sequence (5’-3’)** | **References** |
| --- | --- | --- | --- |
| *Bifidobacterium* | g-Bifido-F | CTCCTGGAAACGGGTGG | Matsuki et al., 2002(57) |
|  | g-Bifid-R | GGTGTTCTTCCCGATATCTACA |  |
| *Lactobacillus* | LbLMA1-rev | CTCAAAACTAAACAAAGTTTC | Massi et al., 2004(58) |
|  | R16-1 | CTTGTACACACCGCCCGTCA |  |
| *L. gasseri* | Lgas-3 | AGCGACCGAGAAGAGAGAGA | Song et al., 2000(59) |
|  | Lgas-2 | TGCTATCGCTTCAAGTGCTT |  |
| *A. muciniphila* | AM1 | CAGCACGTGAAGGTGGGGAC | Collado et al., 2007(60) |
|  | AM2 | CCTTGCGGTTGGCTTCAGAT |  |

Table 1. Oligonucleotide sequences for real-time PCR amplification
